# Detection of methylation in the CpG islands of the p16INK4A, RASSF 1A and methylguanine methyltransferase gene promoters in pancreatic adenocarcinoma

**DOI:** 10.3332/ecancer.2009.131

**Published:** 2009-01-15

**Authors:** D Paikos, P Stravoravdi, S Voyatzi, I Boukovinas, P Papakotoulas, A Kiziridou, G Sibilidis, I Stergiou

**Affiliations:** 1 Gastroenterology-Oncology Department, Theagenio Cancer Hospital, Thessaloniki, Greece; 2 Research Department, Theagenio Cancer Hospital, Thessaloniki, Greece; 3 2nd Oncology Department, Theagenio Cancer Hospital, Thessaloniki, Greece; 4 Pathology Department, Theagenio Cancer Hospital, Thessaloniki, Greece; 5 Surgery Department, Theagenio Cancer Hospital, Thessaloniki, Greece

## Abstract

Pancreatic cancer consists of an accumulation of genetic and epigenetic alterations. Recently, aberrant methylation of CpG islands of cancer-related genes has emerged as an important epigenetic mechanism of their transcriptional dysregulation during tumour development [1]. Therefore, new diagnostic methods, for early detection based on a better understanding of the molecular biology of pancreatic cancer, are required. We examined the methylation status of p^16INK4A^, RASSF 1A and methylguanine methyltransferase (MGMT) genes considered to be inactivated by promoter methylation in several tumours.

The p^16INK4A^ is an important G1/S cell cycle regulator gene [2]. RASSF 1A gene is involved in apoptotic signalling, microtubule stabilization and cell cycle progression [3]. The MGMT gene removes mutagenic and cytotoxic alkyl-adducts from the O6-position of guanine in DNA. Hypermethylation of the gene leads to the inactivation of DNA repair and to microsatellite instability [4].

To date, little is known about the exact role of hypermethylation of these genes in pancreatic adenocarcinoma, as the molecular mechanisms underlying these neoplasms remain poorly understood.

## Materials and methods

Tumour specimens from 13 patients with pancreatic adenocarcinoma (male/female: 8/5, mean age 67.2 years, range 54–79) were evaluated for the methylation status of p^16INK4A^, RASSF 1A and MGMT genes.

Genomic DNA was extracted from formalin-fixed paraffin-embedded tumour tissues.

DNA methylation was determined by chemical modification of genomic DNA with sodium bisulfite and subsequent a double ‘hot start’ methylation-specific polymerase chain reaction (MSP). MSP has been designed to distinguish methylated from unmethylated DNA. ‘Hot start’ polymerase chain reaction (PCR) permits the Taq polymerase to begin the reaction only after the template and primers are in single-stranded form.

All double MSPs were performed with controls for unmethylated and methylated alleles and a control with H_2_O. Ten microlitres of each second MSP reaction were directly loaded onto 2% agarose, stained with ethidium bromide and visualized under UV illumination. The presence of a visible MSP product in those lanes marked M indicated the presence of methylated alleles. The results on promoter methylation of all genes were correlated with known clinicopathological parameters.

## Results

**Figure 1:**
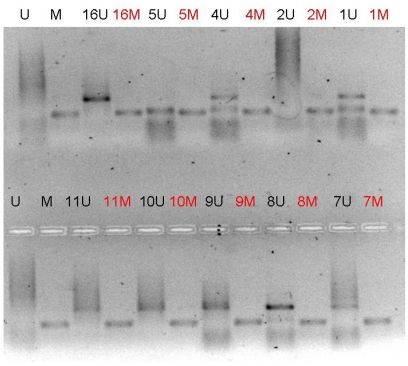
MSP analysis of p^16INK4A^ on agarose gel. All samples were run with both PCR products for unmethylated and methylated portions. The presence of a visible PCR product in these lanes marked U indicates the presence of unmethylated alleles; the presence of product in these lanes marked M indicates the presence of methylated alleles. In this electrophoresis, all patients presented hypermethylation of p^16INK4A^ promoter region. Primer set for U p^16INK4A^: Sense primer 5′-TTATTAGAGGGTGGGGTGGATTGT-3′, Antisense primer 5′-CAACCCCAAACCACAACCATAA-3′. Primer set for M p^16INK4A^ : Sense primer 5′-TTATTAGAGGGTGGGGCGGATCGC-3′, Antisense primer 5′-GACCCCGAACCGCGACCGTAA-3′.

**Figure 2:**
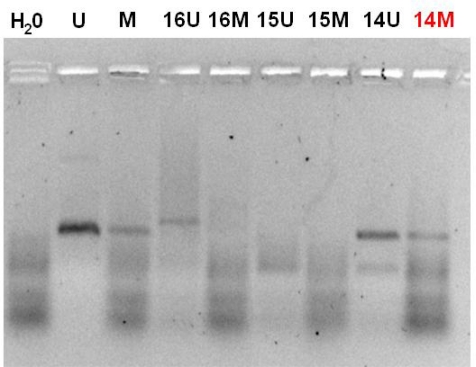
MSP analysis of O6-MGMT gene. All samples were run with both PCR products for unmethylated and methylated portions. The presence of a visible PCR product in these lanes marked U indicates the presence of unmethylated alleles; the presence of product in these lanes marked M indicates the presence of methylated alleles. In the present electrophoresis, the patient in lane 14M presented hypermethylation of O6-MGMT promoter region. Primer set for U O6-MGMT: Sense primer 5- TTTGTGTTTTGATGTTTGTAGGTTTTTGT-3′, Antisense primer 5′-AACTCCACACTCTTCCAAAAACAAAACA-3′. Primer set for M O6-MGMT: Sense primer 5′-TTTCGACGTTCGTAGGTTTTCGC-3′, Antisense primer 5′-GCACTCTTCCGAAAACGAAACG-3′.

The p^16INK4A^ gene was methylated in all 13 patients (Figure 1). RASSF 1A was methylated in two patients, while the MGMT gene was methylated only in the patient with poorly differentiated pancreatic adenocarcinoma (Figure 2). The same patient has also methylated and the RASSF 1A gene. Lymph node status did not correlate with methylation of gene promoters in pancreatic adenocarcinoma patients.

## Conclusions

MSP provides a positive, sensitive (detection 1 methylated allele in a background of 1000 unmethylated alleles), quick and cost-effective test to analyse methylation status of CpG dinucleotides in a CpG island, making the technique suitable for high-throughput analysis of clinical samples. Our findings suggest that p^16INK4A^ gene is commonly down-regulated in pancreatic adenocarcinoma through a promoter hypermethylation of CpG islands. Of course, the small group of 13 analysed patients does not allow us to support the exact role of hypermethylation of RASSF 1A and/or MGMT on pancreatic cancer, yet. To this point, further studies with more patients are now carried out in our lab in order to assess if these epigenetic alterations provide additional information regarding to pancreatic adenocarcinoma prognosis and finally offer additional parameters for potential biological therapeutic strategies of this aggressive disease.
